# New Form of Antiseptic for Local Use

**Published:** 1867-06

**Authors:** 


### New Form of Antiseptic for Local Use.

*The liquor carbonis detergens* is recommended. It is an alcoholic solution of coal-tar, containing, we presume, the carbolic, phenic, and other acids, with dark tarry matter, and differing from carbolic acid, as the liquor cinchonæ does from quinine. It readily mixes with water, forming a permanent emulsion, and in various strengths is available as a mouth-wash, a gargle, an injection for fetid uterine discharges, cancer, retained placenta, etc., gonorrhœa in the female, foul ulcers, sloughing sores, and all maladies dependent in, or complicated by, parasite beings, lice, fungi, etc. It is also used combined with soda.—*Medical Times and Gazette.*

